# Young smokers and non-smokers perceptions of typical users of plain vs. branded cigarette packs: a between-subjects experimental survey

**DOI:** 10.1186/1471-2458-13-1005

**Published:** 2013-10-24

**Authors:** Ingeborg Lund, Janne Scheffels

**Affiliations:** 1 Norwegian institute for alcohol and drug research (SIRUS), PO.box 565Sentrum, Oslo N-0105, Norway

**Keywords:** Plain packaging, Adolescents, Survey, Smoking

## Abstract

**Background:**

In an attempt to minimize the pack design avenue of communication between tobacco producers and smokers and potential smokers, several jurisdictions, including Norway, have considered regulations on cigarette pack design. The main aim of the current study was to investigate how package design affects young people’s perceptions of typical smokers of some pre-chosen cigarette brands and brand varieties.

**Methods:**

Based on data from a web survey among 1022 15–22 year-olds, possible effects of plain packaging of cigarettes on adolescents’ views about typical cigarette smokers were investigated. The data collection had a between-subjects design, in which participants were allocated to one of three groups, and asked to typify the smokers of selected cigarette packs either in branded, plain or plain with descriptor versions. The sample included boys and girls, and smokers and non-smokers. The smoker characteristics included in the investigation were: gender, glamour, stylishness, popularity, coolness, sophistication and slimness.

**Results:**

After creating sum-scores within and across packs and pack versions, analyses indicated that a shift from branded to plain cigarette packaging would result in a reduction in positive user images related to smoking among adolescents and young adults. For girls, this effect held up after controlling for confounders.

**Conclusions:**

To the extent that plain packaging contributes to making smoking images less positive, it can potentially be an efficient aid in reducing smoking uptake among adolescents.

## Background

Smoking is a social behaviour, entangled in cultural meanings and implicit understandings. Structural factors, such as legislative measures and a steadily more negative norm climate [1-3] have probably influenced opinions about the practice itself, as well as ideas about the archetypal smoker. Today, regular adult smokers tend to be seen in a negative light, with connotations of poor health, low education and other psychosocial problems [1,4]. Among adolescents, however, this image is less distinct. Studies of the social meaning of smoking in the age group in which smoking uptake usually occurs have shown that negative images of smoking blend together with associations to maturity or success [3]. Young people tend to be more influenced by counter-cultural currents, possibly resulting in a tendency to react contrary to anti-smoking conventions. Furthermore, leeway offered by a perceived undetermined social position probably diminishes the effect of negative norms [5], while a tendency towards an overly optimistic outlook on the personal ability to quit [6] can make the decision to smoke more trivial.

Research has shown that earlier smoking onset increases the risk for high nicotine dependence later on [7], and to reduce the recruitment of new smokers among youth is one of the most important goals of anti-tobacco work everywhere.

In Norway, anti-tobacco work has long traditions, and is largely based on judicial restrictions. A complete ban on tobacco advertising has been effective since 1975, indoor smoking in public areas and work places was made illegal in 2004, and in 2010 a point of sale tobacco display ban was introduced. The legal age for purchasing tobacco was raised from 16 to 18 years in 1996. Combined with consistently high tobacco tax levels, these restrictions have probably made important contributions to the reduction in daily smoking prevalence from more than 40 per cent of adults in the 1970s, to 17 per cent in 2011 [8]. For adolescents, daily smoking prevalence was 25 per cent for boys and 29 per cent for girls in 1986 (15–20 year-olds) [9], but only 7 per cent in 2012 (16–24 year-olds) [8].

Referring to associations made between the users of a particular brand and the identities and personalities of the brand’s image, cigarette packs have been described as “badge products” [10]. Lacking alternative means of communication in a situation where traditional advertising is illegal, the design of cigarette packs have gained importance and become one of the central factors in the promotion of tobacco products [11,12] functioning to attract customers, create ideas about user characteristics [13], and foster brand loyalty [12-14]. Designing cigarette packs to appeal to different categories of consumers is part of this strategy, and research have shown that, based solely on the design of the packs, young adults will ascribe different attributes and images to cigarette brands even if they are not familiar with the brand [15]. The differentiation of the designs of “feminine” and “masculine” products is an important dimension of brand image building, as disclosed by several studies [16,17]. The tobacco industry has also made deliberate efforts to create designs that attract young smokers [10,12], and it has been shown that cigarette brand imagery and symbolic properties used to create social personas to communicate to peers are particularly valued by adolescents [18,19].

In addition to colouring and illustrations, variant descriptors play a crucial role in the designs of cigarette packs, making it possible to discriminate between different variants within brands. As identified by research, a problematic aspect of variant descriptors is that they function to create erroneous ideas about less harmful types of cigarettes [20,21]. In several jurisdictions, including Norway, this has led to the ban of misleading descriptors like “mild” or “light”, although it has become increasingly clear that smokers still hold erroneous beliefs about relative harm based on still-existing descriptors such as “smooth” or the names of colours [22], particularly when combined with other design features for example the use of a lighter coloured pack or slimmer cigarettes [23,24]. In Norway it is common to associate light pack colours with female smokers, while darker pack colours more often are seen to indicate variants more used by men [18], and it is likely that this applies also to colour descriptors, making “gold” or “light blue” more feminine than e.g. “red” or “black”. Other variant descriptors typically found in the Norwegian market might create ideas eco-friendliness (no additives), better taste (rich), or traditionality (original).

In an attempt to minimize the pack design avenue of communication between tobacco producers and smokers and potential smokers, several jurisdictions have considered regulations on cigarette pack design. Recently, as the first jurisdiction worldwide, plain packaging was introduced in Australia [25]. Results from experimental studies in several countries indicate that introducing plain packs can have large implications for how young people view cigarette brands. Generally, adolescents and young adults tend to ascribe fewer positive characteristics to users of plain packs [26,27], and are also themselves less attracted to specific brands when presented as plain packs [27-30]. A reduced tendency to believe that some cigarettes are less harmful than others have been reported [31], and several findings have indicated that plain packs might encourage smoking cessation or reductions [16,32]. Furthermore, increased visual attention to health warnings can make the harmfulness of cigarettes more salient, particularly to non-smokers and less-than-daily smokers [33].

Norway have ratified the WHO Framework Convention on Tobacco Control where article 11 states that the authorities “should consider adopting measures to restrict or prohibit the use of logos, colours, brand images or promotional information on packaging other than brand names and product names displayed in a standard colour and font style (plain packaging)”. The potential impact of plain packaging in the Norwegian context has previously been explored in qualitative studies, which have shown how package design affects identification with and differentiation between brands [18] and how plain packaging seems to affect these processes [34].

The main aim of the current study was to investigate how package design affects young people’s perceptions of typical smokers of some pre-chosen cigarette brands and brand varieties. It has been shown that a positive view of typical smokers is associated with a higher risk of relapse for former smokers [35]. Peer pressure and acceptance is paramount for teenagers, and cigarette brand and smoker images might be correspondingly important. Positive prototype ideas in this group are associated with a higher risk of becoming a smoker [36], while negative views are associated with a lower tendency to experiment with smoking [37].

## Methods

This study was approved by the Norwegian Social Science Data Service (NSD), reference number 25545, and adheres to the national ethics guidelines [38]. In a web-survey conducted in the spring 2011, 24 different packs of cigarettes were shown in branded or plain versions to 1022 15–22 year old smokers and non-smokers who were asked to indicate which characteristics they thought were typical for their users. The participants were recruited from TNS Gallup’s online participant panel. All cigarette varieties included in the study were purposely selected from leading international and Scandinavian brands to reflect key dimensions of interest in terms of brand descriptors and imagery. This included the selection of brands and variants that featured different colour or flavour descriptors, and the inclusion of packs that featured different brand imagery, e.g. different colours, as well as packs in different sizes (10s and 20s). The market was dominated by the Scandinavian brand Prince, and Marlboro (in different brand varieties), with market shares of 39 and 24 per cent, respectively. The next most popular brands were Kent (market share 6%) and Lucky Strike (5%), while the budget brands Paramount and West both had market shares of about two per cent, and Petteroes cigarettes had a one per cent share [39].

Packs distinctly targeted at female consumers, such as Vogue or 'Slims’ type cigarettes (often described as “glamour packs”), are rare in Norway, and such packs were therefore not included in the pack selection. Nevertheless we aimed at including packs that had a pronounced gender profile, and selected those available in the Norwegian market at the time of the interview that most clearly fit this description (e.g. Marlboro Gold, Kent Surround system).

### Design

**Figure 1:**
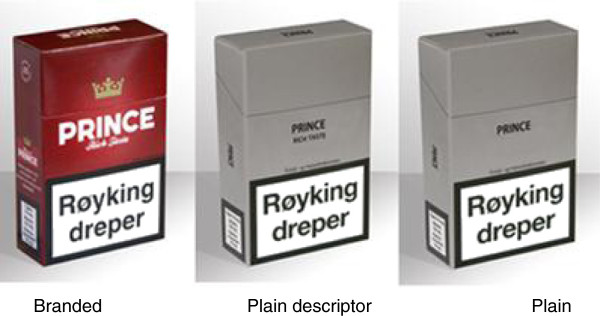
Examples of the three versions of cigarette packs.

The data collection had an experimental, between-subjects design, in which participants were allocated to one of the three pack versions. Individual pictures of cigarette packs were shown to three separate groups of respondents in a branded, a plain, or a plain with descriptor version, as illustrated in Figure 1. To avoid systematic bias depending on where in the questionnaire a pack was positioned, the packs were randomly ordered between participants. As adolescents tend to view some brand varieties as “male” and others as “female” [18], boys and girls were shown two different pack selections.

The number of packs was larger than the number of brands in this study. To avoid the situation were plain pack versions of two brand variants were completely identical, it was decided to use alternative colours on 6 plain packs. However, with regard to interpreting differences in average views on plain and branded packs this posed a potential problem, and for the purpose of this study it was decided to leave these 6 packs out. The current analyses are thus based on 9 different packs within each gender (Additional file 1: Table S1). The branded packs were purchased in regular shops, while images of the plain and plain with descriptor versions were designed specially for this project. All the text was written in the same generic font on all the plain packs and the plain with descriptor packs.

Additional information from the survey included the respondents’ views on tobacco smoking, health risks from tobacco smoking and tobacco regulations, demographic background variables and personal smoking habits.

There were no statistically significant differences in the age, gender or smoker distributions of the participants according to type of pack. Overall 41.8 per cent of the participants were male, and there were 79.5 per cent non-smokers in the total sample. Regarding the age distribution, 16.6 per cent were 15–17 year-olds, 44.7 per cent were 18–20 year-olds, and 38.7 per cent were 21–22 year-olds.

### Measures

Seven user-characteristic indexes (sum-scores) were calculated based on the respondents’ classifications of typical users of each type of cigarette according to the dimensions gender, glamour, style, popularity, coolness, sophistication, and slimness. All original variables had three response categories (users typically possess the quality; typically do not possess the quality; the quality has no bearing on the choice of this cigarette), and sum-scores were calculated in a two-step procedure starting with dichotomizing all variables (1 = possesses; 0 = does not possess, or has no bearing).

Gender-specific factor analyses (principal component) on the user-characteristic indexes indicated that all seven indexes were part of a single construct (single factor solutions; KMOs =0.90; 0.87, α = 0.89; 0.88), and therefore could be joined together. This led to the construction of two additional types of indexes. First, single-pack dummies were created by adding together the seven dichotomized user characteristics for each pack, before dichotomizing into those who gave at least one positive user characteristic vs. those who gave none. The background for applying this strict criterion was that descriptive inspection of the data revealed that the majority of the respondents gave zero or one positive user characterisations for each pack. While the proportion who gave no positive user characteristic ranged from 34.5 to 60.1 per cent among boys, and 40.1 to 79.2 per cent among girls, the corresponding proportions who gave one positive characteristic ranged from 16.4-47.2 per cent and 11.1-19.8 per cent for boys and girls respectively. Consequently only a relatively small proportion of the respondents assigned 2 or more positive characterisations to each pack. Second, to facilitate a global linear regression analysis, a global sum-score was calculated by adding together all binary categories across all brands, pack versions and user characteristics.

Additional variables to use in analyses were age (coded into three age groups), gender and smoking status. Smokers were defined as those who had smoked at all during the last 30 days. Attitudes to smoking and smoking regulations might potentially influence respondents’ perceptions of cigarette packs and smokers. In regression analyses, five different attitudes, measured prior to exposure to cigarette pack images, were included as dummy variables: “society is negative to smoking”, “smoking helps smokers stay slim”, “smoking makes you addicted”, “a cigarette every now and then is not damaging to health”, and “cigarette packs ought to have more information about health consequences”. There were significantly more boys in the group exposed to plain pack versions who thought that smoking helps smokers stay slim (Chi square, p < 0.01), but otherwise there were no significant differences between groups regarding these attitudes.

### Analyses

Unadjusted ORs for giving at least one positive user characteristic were estimated based on 18 single-pack dummy variables. Within each gender, averages of the seven user-characteristic indexes across cigarette makes were calculated, and significant differences between pack versions were tested using Anova with Bonferroni post hoc tests. Gender-specific linear regressions on the global index, with pack version as an independent variable, controlling for age, smoking status, and attitudes, were performed.

## Results

Additional file 1: Table S1 shows the percentages of respondents who gave at least one positive user characteristic for each branded cigarette pack, and the unadjusted odds ratios (ORs) for this to occur for branded packs vs. plain versions of packs. For added simplicity, and as there were small differences between them, the two plain pack versions were collapsed in these analyses.

For boys, the OR of giving a positive score was significantly higher in the branded version of five makes, while there was no significant difference associated with pack version for the remaining makes. For girls, the OR of giving a positive score in the branded version was significantly higher for three makes, significantly lower for three makes, and not significantly different for three.

Generally, the proportions of participants who assigned at least one positive user characteristic to the branded pack versions differed a lot according to make, for both genders. For boys, this share ranged from 44.7 per cent (Paramount Red American Blend) to 71.5 per cent (Petteroes Original). For girls, the allotment of positive scores ranged from 23.0 per cent (Paramount Red American Blend) to 73.9 per cent (10 Marlboro gold).

Turning to the user-characteristics indexes (Table 1), bivariate comparisons showed one significant difference in how boys exposed to the plain and boys exposed to the branded versions of packs viewed the users of these cigarettes, namely that boys exposed to the branded version more often thought that the users were typically boys (p < 0.05). There were no significant differences between the boys’ rating of users in the plain with descriptor vs. the branded version, or between the two plain versions.

**Table 1:** Average user-characteristic index scores for the three pack versions, boys and girls

	**Branded**^**a**^	**Plain descriptor**^**b**^	**Plain**^**c**^	***Group sizes (N)***		
				***Branded***	***Plain descriptor***	***Plain***
Boys						
Boy	3.0	2.8	2.5*	*156*	*112*	*141*
Glamorous	1.4	1.5	1.3	*153*	*110*	*136*
Stylish	1.9	1.6	1.5	*153*	*111*	*135*
Popular	1.4	1.5	1.2	*152*	*111*	*139*
Cool	1.5	1.4	1.1	*151*	*111*	*140*
Sophisticated	1.1	1.3	1.2	*150*	*111*	*140*
Slim	1.5	1.4	1.4	*155*	*111*	*136*
Girls						
Girl	2.6***	1.7	2.0**	*208*	*185*	*161*
Glamorous	1.7***	1.0	1.2**	*204*	*187*	*157*
Stylish	1.9***	1.1	1.2***	*209*	*185*	*159*
Popular	1.4*	0.9	1.1	*204*	*181*	*160*
Cool	1.3	1.0	1.1	*202*	*184*	*160*
Sophisticated	1.5***	0.9	0.9**	*201*	*183*	*159*
Slim	1.3	0.9*	1.4	*208*	*184*	*157*

Anova with Bonferroni post-hoc test, *:p < 0.05, **:p < 0.01, ***:p < 0.001.

^a^ star signifies significant difference between branded & plain descriptor versions.

^b^ star signifies significant difference between plain descriptor & plain versions.

^c^ star signifies significant difference between branded & plain versions.

Girls exposed to branded packs significantly more often than girls exposed to plain or plain descriptor packs responded that the typical pack user was a girl (p < 0.001 and p < 0.01), glamorous (p < 0.001 and p < 0.01), stylish (both p < 0.001), and sophisticated (p < 0.001 and p < 0.01) (Table 1). Furthermore, compared to girls who were shown the plain with descriptor version, those who were shown branded packs gave a significantly higher score on the user-index popular (p < 0.05). The answers for the two plain pack versions differed significantly for the user characteristic “slim” (p < 0.05), with the lowest score given for the plain with descriptor version of the packs.

In linear regression analyses (Table 2), pack design significantly influenced the total number of positive user characteristics given by girls, but not by boys. On average, for girls exposed to branded packs the global index score was 3.4 points higher than the score for girls exposed to plain packs.

**Table 2:** Linear regressions of the effect of type of pack on the global index score for boys and girls

	**Girls**^**a**^	**Boys**^**a**^
Constant (ref. person)	7.4***	10.1***
Branded condition	3.4***	1.6
Smoker	1.7*	0.7
Age Group (ref: 15–17 years)		
18-20 years	0.4	-0.4
21-22 years	-1.8	-1.4
Attitudes		
Society is negative to smoking	0.8	1.1
Smoking helps smokers to stay slim	1.8	4.7***
Smoking makes you addicted	-0.5	-1.7
To smoke a cigarette every now and then is not damaging to health	2.3*	1.4
Cigarette packs should have more information about health consequences	0.3	1.0
*Adj. R-square*	*0.071*	*0.033*

^**a**^: Unstandardized coefficients. The constant is the calculated sum-score of the reference person. The reference person is a 15–17 year-old non-smoker exposed to non-branded packs, with attitudes opposite of those listed in the table.

t-test, *:p < 0.05, ***:p < 0.001.

For boys, the comparable (non-significant) difference in global index scores between branded and non-branded versions of packs was 1.6 points. Additionally, girls who were smokers and girls who did not think that the occasional cigarette was damaging to their health, also had a significantly higher tendency to give positive user characteristics. For boys, the only significant variable was to believe that smoking helped people to stay slim, but in return the effect of that attitude was quite strong, giving an average increase in the general index of 4.7 points. Similar linear regressions on each of the individual characteristics listed in Table 1 (not reported in the table) showed significant effects of pack version on the scores for all characteristics except slimness for girls. For boys, pack version had a significant effect on the scores for gender, stylishness and coolness, but not for the other aspects. Consistent with the results reported in Table 2, the attitude that a cigarette every now and then is not damaging to health significantly increased the index score for several of the characteristics for girls, while the attitude that smoking helps smokers to stay slim significantly increased the scores for own gender and slimness. For boys, the attitude that smoking helps smokers to stay slim significantly increased the score for own gender, popularity, stylishness, coolness and slimness.

## Discussion

In this study, pack design had implications for the participating adolescents’ and young adults’ views of prototype smokers of different brands. First of all, the large variability in how different brands were evaluated indicates that cigarette packaging is important for young people’s differentiation between, and possibly also identification with, brands. Second, exposure to branded packs generally resulted in a more positive characterization of smokers both within and across makes. For each make, both genders had a stronger tendency to give a least one positive user characteristic for the branded vs. the plain version of the pack. Across makes, the branded version of the packs stood out with a higher occurrence of almost all of the positive user traits and a higher global user characteristic for girls, an effect that persisted after controlling for confounders. The difference in user characterisations between branded and plain pack versions was less striking for boys, who nevertheless were more likely to think that the users of the branded packs were boys.

These results indicate that a shift from branded to plain cigarette packaging might lead to a reduction in positive images related to smoking among adolescents and young adults. According to the theory of symbolic consumption, positive images tied to smoking and specific brands allow users to create identities through their smoking that they project to others [13,40]. Identical packaging for all brands would make it more difficult to signal affinity to any particular sub-group of smokers, making any cigarette much more a mere deliverer of nicotine. To the extent that plain packaging contributes to making smoking images less positive, it can potentially be an efficient aid in reducing smoking uptake among adolescents.

One of the most striking aspects of the results from this study was the dissimilarity in the responses from boys and girls. There were many significant differences in the girls’ user characterisations associated with pack version. In contrast, boys demonstrated more stable ideas regardless of which version they were shown, and in the regression analysis, pack version had no significant influence on the user characteristic score for boys. One interpretation is that this indicates that pack design is less important for how boys view typical smokers, and that boys are just less interested in, and therefore less influenced by, the design of cigarette packs. However, it is also possible that this result reflects the greater efforts made by the tobacco industry to design cigarette packs more palatable for girls [17]. The idea that females are more influenced by pack design, either because of a larger array of “female” designs, or due to other factors, is not novel within this research field, and some of the previous research in fact included only girls [16,27,29,30]. However, previous results regarding the significance of gender on associations made between pack designs and perceived attributes, can also be said to be conflicting. Recently, no significant gender differences were found in a study of young adults’ evaluation of different tobacco brands [14], while another study found a gender difference related to a particularly feminine pack, but no gender difference in the associations between plain pack colour and appeal [41].

On the other hand, looking particularly at the single pack analysis, the difference between boys and girls becomes less pronounced. Within both genders there were several significant differences in the proportions who assigned positive user characteristics in the branded and plain version of individual packs. Moreover, while boys in all significant situations rated the branded version of the pack more positively than the plain, girls had a more diversified reaction, with some branded packs actually being rated significantly less positive than their plain counterparts. The fact that the male reactions did not lead to many significant differences across packs might suggest a larger degree of individual variation within this group.

A potential problem in the interpretation of the lack of design-reactions among boys is that the user characteristics asked for in this study probably were tapping feminine dimensions more, and as such were less appropriate for boys. It has been shown that factors related to a positive individual competence prototype (e.g. smart, independent, good-looking, considerate) are important in influencing lifetime smoking prevalence for boys [37], and this dimension was largely missing from the current study (with the possible exception of “slimness”).

Another limitation in this study is that all respondents would have been quite familiar with the design of the branded cigarette packs, and had preconceived ideas about prototypical users before they participated in the study. The likelihood of this was increased by the fact that the selected range of packs consisted of brands and varieties that were all quite popular and well known. However, if respondents let former prototype ideas influence their answers to the plain version of the packs, it is likely that this would have worked to reduce the difference between responses to the branded and plain packs compared to the situation where everybody was neutral from the start.

The between-subject design also creates some challenges, primarily the risk of uncontrolled variation between groups, or in this instance, between versions of packs. Fortunately, the groups did not differ statistically from each other in terms of age or smoking status. However, it is feasible that the bivariate lack of difference between the responses of boys to the plain and branded pack was associated with the significantly larger proportion of participants in the plain pack groups who thought that smoking helped smokers to stay slim. It is of course also possible that other factors that were not measured could have influenced the variation found between groups.

It is not unlikely that introducing plain packaging might have some unintended effects that could undermine the effect of the policy. Smokers may decide to import more cigarettes legally or illegally, producers of cigarette cases could potentially see plain packaging as a new business opportunity, and the tobacco industry may counteract the similarity of the packs by changing the design of the cigarettes. However, most of these effects would in all likelihood be short term, while the positive effects of plain packaging could be expected to increase as both smokers and potential smokers start to put their outmoded ideas about user characteristics behind them in the medium to long term.

## Conclusions

Plain cigarette packaging is associated with a reduction in positive images related to smoking among adolescents and young adults. This is an effect that could be expected to become stronger in the long term. To the extent that plain packaging contributes to making smoking images less positive, it can potentially be an efficient aid in reducing smoking uptake among adolescents.

## Competing interests

The authors declare that they have no competing interests.

## Authors’ contributions

IL performed the statistical analyses and drafted the manuscript. JS designed the study and helped to draft the manuscript. Both authors read and approved of the final manuscript.

## Pre-publication history

The pre-publication history for this paper can be accessed here:

http://www.biomedcentral.com/1471-2458/13/1005/prepub

## Supplementary Material

Additional file 1: Table S1 Unadjusted OR for giving at least one positive user characteristic for branded vs. plain packs. Click here for file
