# Supplementary figures and images for: Young smokers and non-smokers perceptions of typical users of plain vs. branded cigarette packs: a between-subjects experimental survey
Source: BMC Public Health. 2013 Oct 24;13:1005. doi: 10.1186/1471-2458-13-1005 (PMC4015779; doi:10.1186/1471-2458-13-1005)

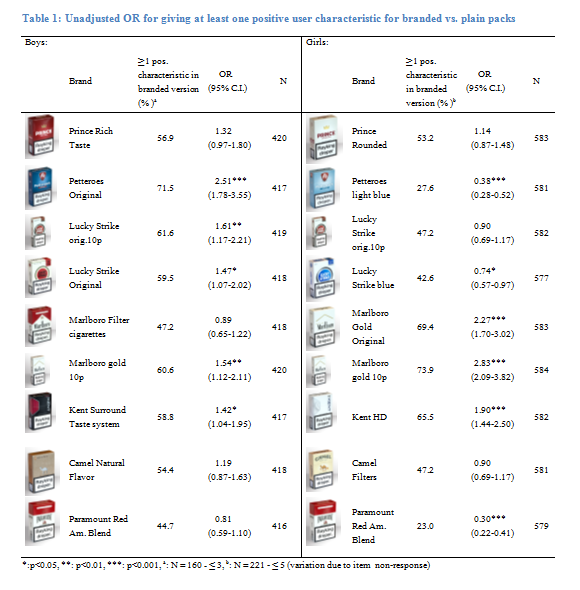

Supplement: Additional file 1: Table S1 — Unadjusted OR for giving at least one positive user characteristic for branded vs. plain packs. [file 1471-2458-13-1005-S1.PNG]
